# Laser Fabrication of Bioinspired Graphene Surfaces With Superwettability

**DOI:** 10.3389/fchem.2020.00525

**Published:** 2020-06-23

**Authors:** Zhuo-Chen Ma, Chun-He Li, Xin-Yu Hu, Bing Han, Yong-Lai Zhang, Qi-Dai Chen, Hong-Bo Sun

**Affiliations:** ^1^State Key Lab of Precision Measurement Technology and Instruments, Department of Precision Instrument, Tsinghua University, Beijing, China; ^2^State Key Laboratory of Integrated Optoelectronics, College of Electronic Science and Engineering, Jilin University, Changchun, China

**Keywords:** laser fabrication, graphene, graphene oxide, bioinspired surfaces, superwettability

## Abstract

The past decades have seen growing research interest in developing efficient fabrication techniques for preparing bioinspired graphene surfaces with superwettability. Among the various fabrication methods, laser fabrication stands out as a prominent one to achieve this end and has demonstrated unique merits in the development of graphene surfaces with superwettability. In this paper, we reviewed the recent advances in this field. The unique advantages of laser fabricated graphene surfaces have been summarized. Typical graphene surfaces with superwettability achieved by laser fabrication, including superhydrophobic graphene surfaces, oil/ water separation, fog collection, antibacterial surfaces, surface enhanced Raman scattering (SERS), and desalination, have been introduced. In addition, current challenges and future perspectives in this field have been discussed. With the rapid progress of novel laser physical/ chemical fabrication schemes, graphene surfaces with superwettability prepared by laser fabrication may undergo sustained development and thus contribute greatly to the scientific research and our daily life.

## Introduction

Inspired by the intriguing superwetting phenomena existed in nature, such as lotus leaf, rice leaf, butterfly wing, and water strider legs, considerable efforts have been devoted to the development of bioinspired surfaces with superwettability including superhydrophilic (water contact angle (WCA) close to 0°), superhydrophobic (WCA > 150°), superoleophilic (oil contact angle (OCA) close to 0°), and superoleophobic (OCA > 150°) surfaces (Tian et al., 2014; Wang et al., 2015b). Moreover, when the surfaces are put in water or oil, other kinds of superwettability may emerge, e.g., underwater superoleophobic, underwater superoleophilic, underoil superhydrophobic, and underoil superhydrophilic surfaces (Su et al., 2016; Liu et al., 2017a). Currently, many techniques have been successfully proposed to fabricate high contrast wettability pattern, such as photocatalytic reaction, microcontact printing or inkjet printing (Gleiche et al., 2000; Tawfick et al., 2012; Lai et al., 2016; Liu et al., 2019). Thus far, bioinspired surfaces with superwettability have revealed great potential for cutting-edge applications in anticorrosion, anti-icing, drag reduction, self-cleaning, and water harvesting (Liu et al., 2017a; Yang et al., 2019; Wu et al., 2020). Therefore, triggered by these promising applications, researchers have adopted various materials to prepare bioinspired surfaces with superwettability.

Graphene, which features many exceptional properties (high Young's modulus, optical transmittance, flexibility, high chemical/physical stability, and biocompatibility), may have particular merits in superwetting surfaces (Zhang et al., 2012). Up to now, several methods have been successfully developed for the preparation of graphene surfaces, including chemical vapor deposition (CVD) (Wang et al., 2015a), reduction of graphene oxide (GO) (Han et al., 2019, 2020), and solvent exfoliation of graphite (You et al., 2020). All these approaches have demonstrated their unique advantages in the fabrication of graphene or graphene-related surfaces. Nevertheless, the efficient preparation of superwetting surfaces often requires designable patterning, hierarchical micro/nanostructures, and controllable tailoring of the chemical composition simultaneously. However, this is a challenging task for the above-mentioned technologies.

Laser fabrication emerges as a promising technique that can meet these demands. In this mini review, we summarized the recent development of laser fabrication of bioinspired graphene surfaces with superwettability. The unique advantages of laser fabricated graphene surfaces are highlighted. Typical functional graphene surfaces with superwettability produced by laser fabrication are reviewed, including superhydrophobic graphene surfaces, oil/ water separation, fog collection, antibacterial surfaces, surface enhanced Raman scattering (SERS), and desalination. In addition, the challenges and outlook of this field have been discussed briefly.

## Unique Advantages of Laser Fabrication of Graphene Surfaces

Up to now, various laser sources have been adopted for the fabrication of graphene surfaces. Generally, they can be classified into two categories: CW lasers and pulsed lasers. CW lasers can provide continuous output of light, which interacts with graphene or its related materials as a consequence of photothermal effect. While pulsed lasers (e.g., femtosecond and picosecond laser) can interact with graphene-related materials in an ultrafast process, which can reduce the heat-affected zone and thus result in a high resolution (Hu et al., 2019; Lao et al., 2020). Therefore, different laser sources may lead to graphene surfaces with different morphologies and properties, which is beneficial for the production of various functional surfaces. Currently, the main laser fabrication mechanisms of graphene films include laser reduction of GO, laser treatment of CVD graphene, and laser induced graphene from polymers. Generally, the advantages of laser fabrication of graphene surfaces lie in the following three points. Firstly, laser fabrication can enable designable patterning of graphene in a mask-free, chemical-free, and non-contact manner (Ma et al., 2018; Li et al., 2020). From the viewpoint of fabrication techniques, two methods stand out as prominent approaches: two beam laser interference (TBLI) and laser direct writing (LDW) (Ma et al., 2019; Zhang et al., 2019). TBLI fabrication can produce 1D or 2D grating-like microstructures on graphene surfaces in a cost-effective manner (Guo et al., 2012). It can facilitate patterning in the size of cm scale in just several or tens of seconds. Currently, the patterning by TBLI of graphene films is only limited to grating structures, thus hampering its popularization in broader applications. Nevertheless, in the future by using multi-beam laser interference many kinds of periodic lattice structures may be fabricated (Wu et al., 2013). LDW emerges as an alternative approach to satisfying the requirement of arbitrary patterning (Jiang et al., 2018b; Salter and Booth, 2019). By a computer-aided program, the laser writing path, scanning speed and light intensity can be readily controlled, which is versatile for obtaining a wide variety of graphene surfaces as desired (Zhang et al., 2010; Guo et al., 2014). Besides, other lithographic printing methods have also demonstrated their capabilities in designable patterning, such as UV irradiation, photoinitiated surface grafting, inkjet printing, or the combination of these methods (Nakata et al., 2009; Zahner et al., 2011; Lai et al., 2013; Tian et al., 2013). These strategies may further inspire the fabrication of bioinspired graphene surfaces. Secondly, laser fabrication can realize hierarchical micro/nanostructures on graphene surfaces (Jiang et al., 2014). During laser processing of graphene oxide, the removal of oxygen-containing groups (e.g., hydroxyl and carboxyl) can result in nanoscale roughness on the microscale patterning. This is quite important for the superwettability. Thirdly, laser fabrication can tailor the chemical composition of graphene surfaces, which is beneficial for the tuning of surface energy (Guo et al., 2014). Due to the removal of hydrophilic oxygen-containing groups on GO films after laser processing, the surface energy can be reduced significantly. Moreover, since laser fabrication can be implemented in different atmosphere environment, programmable atom-doping can also be achieved during laser treatment of GO films (Guo et al., 2014). By controlling the laser intensities and atmosphere, exquisite control of atom-doping can be realized in selected areas, thus permitting the preparation of complex functional patterns on graphene surfaces. Besides, laser fabrication also allows graphene thinning by controllable etching. Therefore, all these unique advantages contribute greatly to the bioinspired graphene surfaces and promote their applications in various fields.

## Graphene Surfaces With Superwettability Achieved by Laser Fabrication

**Figure 1:**
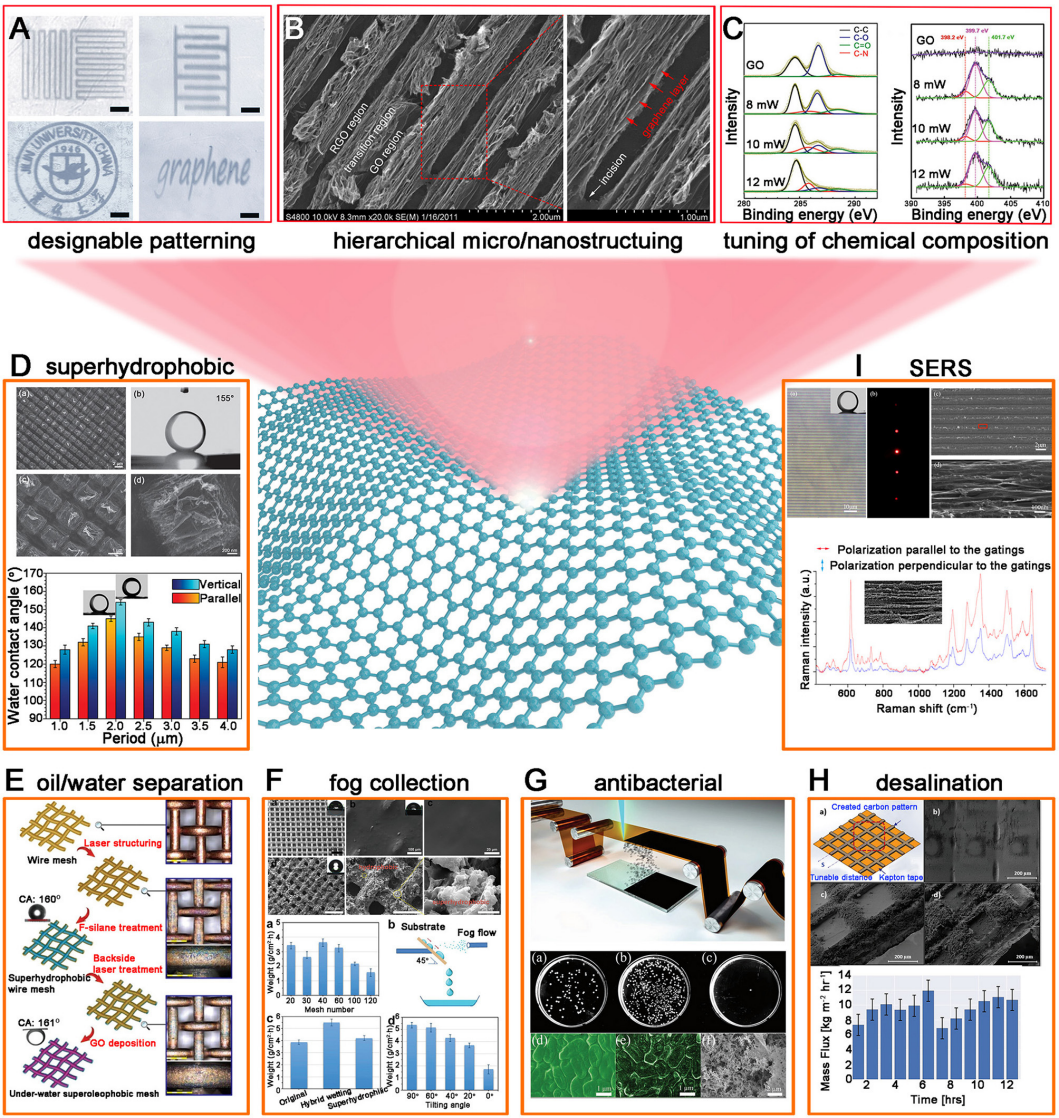
Laser fabricated graphene surfaces with superwettability. **(A)** Designable patterning of graphene films by LDW. **(B)** Hierarchical micro/nanostructuring of graphene films by TBLI. **(C)** Controllable tuning of chemical composition of GO films by laser reduction. **(D–I)** Various graphene surfaces produced by laser fabrication for applications in superhydrophobic graphene surfaces **(D)**, oil/ water separation **(E)**, fog collection **(F)**, antibacterial surfaces **(G)**, desalination **(H)**, and SERS **(I)**. Reproduced from Guo et al. (2014) with permission of WILEY-VCH. Reproduced from Guo et al. (2012) with permission of Elsevier. Reproduced from Jiang et al. (2014) with permission of WILEY-VCH. Reproduced from Liu et al. (2017b) with permission of the Royal Society of Chemistry. Reproduced from Song et al. (2018a) with permission of the Royal Society of Chemistry. Reproduced from Jiang et al. (2020) with permission of WILEY-VCH. Reproduced from Tittle et al. (2018) with permission of WILEY-VCH. Reproduced from Yan et al. (2015) with permission of American Chemical Society.

Due to the above-mentioned prominent advantages of laser fabrication of graphene, it has sparked considerable research interest in developing various functional graphene surfaces for diverse applications in fundamental research and daily life. In this section, we briefly summarized the representative graphene surfaces with superwettability and overviewed their applications (Figure 1).

### Superhydrophobic Graphene Surfaces

Over the past decades, superhydrophobic surfaces (static contact angle, CA > 150°) have been extensively studied due to their promising applications in water droplet transportation (Wu et al., 2011), self-cleaning (Liu et al., 2017a), washable electronics (Das et al., 2017), and wearable elastomer skin (Wang et al., 2018). Inspired by the sliding superhydrophobic lotus leaves with low liquid adhesion and sticky superhydrophobic rose petals with high liquid adhesion, researchers have developed various biomimetic graphene surfaces with controllable wettability and adhesion. Laser fabrication technique serves as an appealing strategy for constructing superhydrophobic graphene surfaces. Wang et al. (2012) innovatively employed TBLI technique to prepare biomimetic graphene surfaces with superhydrophobicity. Due to the hierarchically microscale grating structures with nanoscale roughness and also the removal of hydrophilic oxygen-containing groups, the obtained graphene films exhibit brilliant iridescence with a high CA of 156.7°. Moreover, the biomimetic graphene surfaces demonstrated high adhesion, holding great promise for applications in water transport. Later, to achieve anisotropic superhydrophobicity commonly existed in natural plants (e.g., reed leaf), Jiang et al. (2018a) combined photolithography with TBLI to produce biomimetic graphene surfaces with anisotropic wettability. In their work, microgroove structures (period 200 μm) were fabricated by photolithography and then hierarchical graphene films on the microgrooves were created by TBLI. Consequently, the combined structures demonstrated anisotropic superhydrophobicity. Besides, Jiang et al. (2014) also proposed a dual TBLI fabrication strategy to prepare superhydrophobic graphene films with 2D microscale grating structures. In this manner, the anisotropic hydrophobicity that originates from the 1D grating structures can be avoided. Another technique, LDW, has revealed great potential for its unique advantages in arbitrary patterning capability. Das et al. (2017) adopted LDW to develop superhydrophobic inkjet-printed graphene electrodes for applications in wearable or washable electronics.

In addition to direct processing of graphene surfaces, laser structured templates-supported graphene films serve as an alternative approach to creating superhydrophobic surfaces. Jiang et al. (2016) employed femtosecond laser-structured Cu foil as substrates, on which hierarchical micro/nanostructures were formed after laser writing. Then graphene films with both iridescence and superhydrophobicity could be prepared by CVD on the Cu foil. Besides, biomimetic graphene films with superhydrophobicity and high adhesion can also be prepared on laser structured stainless steel surfaces, which achieved successful transfer of micro-droplets (Song et al., 2017).

### Oil/Water Separation

Currently, oil spillage, industrial wastewater discharge, and emission of edible oil have become a non-negligible problem that seriously threatens the ecological environment and people's health. Therefore, considerable research efforts have been devoted to the development of efficient oil/ water separation surfaces. Liu et al. (2017b) employed femtosecond laser processing and fluorosilane/graphene oxide (GO) modification to fabricate a Janus wire mesh with oil/ water separation capability. Fluorosilane and GO were used for the modification of the two sides of the laser structured mesh, respectively, thus realizing different wettability of the two sides. Consequently, the Janus mesh demonstrated good separation performance of light/heavy oil and water mixtures. Reusability is a crucial issue that needs to be considered for graphene surfaces applied in oil/ water separation. Yu et al. (2019) fabricated graphene surfaces on copper meshes with reversible wettability and self-healing properties by combining poly(dimethylsiloxane) with graphene. The surfaces could be switched between superhydrophilic and superhydrophobic state by using O_2_ plasma etching and laser etching, thus facilitating unidirectional transport of oil or water. Besides, the surfaces showed reversible adhesion. More importantly, the graphene surfaces showed prominent self-healing capability after flame treatment, holding great promise for high-efficiency and reusable oil/ water separation surfaces.

### Fog Collection

Spider nets and desert beetles have demonstrated their unique superiority in fog harvesting, which is significant for their survival in harsh environment. Inspired by this property, Song et al. (2018a) successfully fabricated hybrid polydimethylsiloxane/ graphene surfaces on Cu meshes for fog collection. Because of the distinct hydrophilic and superhydrophobic areas formed by using laser etching and ultrasonic vibration, the hybrid surfaces could drive water droplets toward more wettable regions, demonstrating great potential for fog collection. Besides, they also realized temperature-tunable wettability since the pore size and chemical composition of the laser etched porous graphene surfaces can be tuned by varying temperature (Song et al., 2018b). Moreover, the graphene surface demonstrated high and low adhesion at 0 and 200°C, respectively.

### Antibacterial Surfaces

Antibacterial surfaces are quite important for eliminating antibiotic-resistant bacteria since they can be coated onto various desired substrates. To achieve this goal, Jiang et al. (2020) employed laser induced graphene and laser induced forward transfer to prepare graphene coatings that featured excellent photothermal conversion capability and superhydrophobic property. These two effects synergistically make a great contribution to the antibacterial performance, which can reduce the number of bacteria by more than 99%. Besides, Luong et al. (2019) demonstrated an infiltration method for the fabrication of a robust composite in which laser induced graphene was integrated with various materials. Since the surface roughness can be tuned during laser processing, the composite surfaces exhibited superhydrophobicity and antibiofouling, which could be useful in antibacterial applications.

### Other Functional Surfaces

In addition to the above-mentioned functions, laser fabricated graphene surfaces with superwettability also demonstrate promising applications in other fields, such as SERS and desalination. Yan et al. (2015) fabricated high-efficiency SERS substrates by coating silver films onto the reduced graphene oxide (RGO) gratings that were produced via TBLI. The silver films not only possessed plenty of “hot spots,” but also inherited the superhydrophobic property of the hierarchical RGO gratings, which is beneficial for the enrichment of analytes. Therefore, this effect contributes greatly to the reduction of detection limit. With regard to desalination applications, Tittle et al. (2018) fabricated robust superhydrophobic graphene with a CA of 176°. In this manner, they produced a membrane for water desalination based on air-gapped membrane distillation.

## Conclusion and Outlook

In conclusion, featuring designable patterning, hierarchical micro/nanostructuring, and flexible tuning capability of chemical composition, laser fabrication holds great promise for the fabrication of bioinspired graphene surfaces with superwettability. Typical laser fabricated superwetting graphene surfaces and their applications are summarized in this paper, including superhydrophobic graphene surfaces, oil/ water separation, fog collection, antibacterial surfaces, SERS, and desalination. However, at present, a major challenge lies in that the micro/nanostructures on graphene surfaces may be scratched or even damaged after long term use. In this regard, developing wear-resistant and self-healing graphene surfaces might be a possible solution. In addition, the cost of laser fabrication is relatively high, thus restricting its further applications in industrial manufacturing. Nevertheless, with the rapid development of various laser sources, laser fabrication of superwetting graphene surfaces will find much broader applications in our daily life.

## Author Contributions

All authors listed have made a substantial, direct and intellectual contribution to the work, and approved it for publication.

## Conflict of Interest

The authors declare that the research was conducted in the absence of any commercial or financial relationships that could be construed as a potential conflict of interest.
